# PNRC is a unique nuclear receptor coactivator that stimulates RNA polymerase III-dependent transcription

**DOI:** 10.1186/1750-2187-2-5

**Published:** 2007-07-05

**Authors:** Dujin Zhou, Shuping Zhong, Jing-Jing Ye, Keith M Quach, Deborah L Johnson, Shiuan Chen

**Affiliations:** 1 Department of Surgical Research, Beckman Research Institute of City of Hope, 1450 East Duarte Road, Duarte, CA 91010, USA; 2 Department of Biochemistry and Molecular Biology, Keck School of Medicine and Norris Comprehensive Cancer Center, University of Southern California, Los Angeles, CA 90033, USA

## Abstract

**Background:**

PNRC transcriptionally regulates a wide range of RNA polymerase (pol) II-transcribed genes by functioning as a nuclear receptor coactivator. To search for additional PNRC-interacting proteins other than nuclear receptors, a PNRC fragment was used as bait in a yeast two-hybrid screening of a human mammary gland cDNA expression library.

**Results:**

RNA pol III/RPC39 fragments were repeatedly identified as PNRC-interacting partners in two independent screenings. The interaction between these RPC39 fragments and PNRC was further confirmed in the independent yeast two-hybrid assays. The association of endogenous PNRC and RPC39 in MCF7 cells was demonstrated by co-immunoprecipitation. Furthermore, ChIP analysis detected co-recruitment of PNRC and RPC39 to tRNA and U6 RNA promoters. The biological consequence of the interaction between PNRC and RPC39 was further studied. Overexpression of PNRC, either by transient or stable transfection, increased RNA pol III-dependent transcription in MCF7 cells, while a decrease in transcription in MCF7 cells treated with PNRC/siRNA was observed.

**Conclusion:**

Here, we demonstrate that human PNRC stimulates RNA pol III transcription through its interaction with the subunit RPC39 of RNA pol III.

PNRC is a unique coactivator that has profound effects on many aspects of cellular function by directly influencing both RNA pol II- and RNA pol III-dependent transcription.

## Background

Nuclear receptors are ligand-dependent transcription factors that regulate the expression of various genes by binding to the specific hormone-responsive elements located in the target gene promoters, thus playing essential roles in development, differentiation, cell proliferation, and metabolism. For the past few years, a great deal of progress has been made in understanding the mechanisms by which the nuclear receptors regulate gene transcription. The function of nuclear receptors can be regulated by a number of factors including ligand binding, DNA binding, interaction with other members in the family, interaction with basal transcription factors, and interaction with coactivators and corepressors. Most of these coactivators of nuclear receptors have molecular weights of ~160 kDa and interact with the liganded nuclear receptors using a short hydrophobic motif called NR-box or LXXLL-motif [1].

Our studies to elucidate the mechanisms that regulate the expression of the human aromatase gene in breast cancer have identified and characterized a new family of coactivator proteins, PNRC (proline-rich nuclear receptor coregulatory protein) [2] and PNRC2 [3]. PNRC and PNRC2 were identified as bovine SF-1 (steroidogenic factor 1)-interacting proteins in a yeast two-hybrid screening of a human mammary gland cDNA expression library. PNRC and PNRC2 were found to interact with the ligand-binding domains of all the nuclear receptors tested, including ER, PR, GR, TR, RAR, and RXR, in a ligand-dependent manner. They were also found to interact in a ligand-independent manner with the orphan receptors SF1 and estrogen receptor-related receptor alpha 1 (ERRα1). These coactivators are unique in that they are significantly smaller than most of the coregulatory proteins previously identified and are proline-rich. Unlike most of the coactivators that interact with nuclear receptors through its LXXLL motif, these new coactivators interact with nuclear receptors through a proline-rich Src homology domain-3 (SH3)-binding motif, S-D (E)-P-P-S-P-S [2,3].

In addition to functioning as a coactivator to activate the transcription mediated by multiple nuclear receptors, PNRC was recently found to down regulate the activation of Ras and MAP kinase through interaction with Grb2, an important adapter protein involved in growth factor/Ras signaling pathway [4]. PNRC interacts with two SH3 domains of Grb2 through two SH3-binding motifs at its N- and C-terminus. It is clear that Ras plays a central role in mitogenic signaling. Consequently, inhibition of Ras activation may therefore block the growth of malignant cells that are dependent on activated Ras protein. Our previous data revealed that overexpression of PNRC in HeLa cells suppressed Ras and MAP kinase activation and cell growth [4].

In an attempt to gain insight into the mechanism of transactivation and signal transduction activities of PNRC, we were interested in identifying cellular proteins other than the nuclear receptors that specially interact with PNRC C-terminus, which contains an SH3-binding motif. In this study we employed the Gal4-based yeast two-hybrid system to screen a human mammary gland cDNA expression library with PNRC_270–327 _as bait for the proteins that associate with C-terminal peptide of PNRC. The RNA polymerase III subunit, RPC39, was isolated repeatedly from two independent screenings. Here, we demonstrate specific interaction of RPC39 with PNRC *in vitro *and *in vivo*. Furthermore, our data from functional analysis provide evidence that the interaction between PNRC and RPC39 plays a role in the activation of transcription by RNA polymerase III. Thus, PNRC is a unique coactivator regulating not only the transcription of wide range RNA pol II-transcribed genes by functioning as nuclear receptor coactivator, but also the transcription of genes transcribed by RNA pol III through its direct interaction with a unique subunit of human RNA pol III, RPC39.

## Results

### Identification of RPC39 as PNRC-interacting protein in the yeast two-hybrid screening

**Figure 1:**
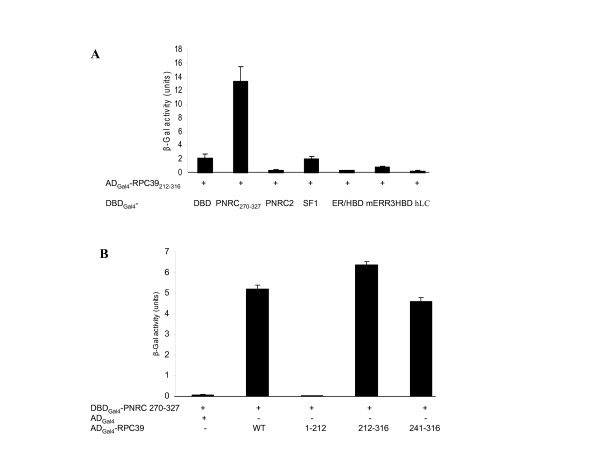
**PNRC interacts with a subunit of RNA pol III**. ***A***, AD_Gal4_-RPC39_212–316 _expression plasmid was isolated from a human mammary gland expression library screening using DBD_Gal4_-PNRC_270–327 _as bait. To confirm the interaction and the specificity of the interaction between PNRC and RPC39, yeast strain Y187 was cotransformed with the expression plasmids for the expression of the fusion proteins as indicated, and transformants containing these plasmids were selected by growth on SD/-Leu/-Trp agar plates. The expression of interacting hybrid proteins in Y187 transformants was analyzed for *Lac*Z expression. DBD_Gal4 _(DBD) and DBD_Gal4_-human lamin C (hLC) expression plasmids were included as background and negative control, respectively. β-Galactosidase activities in liquid cultures are expressed in Miller units as mean ± s.d. of three independent assays. ***B***, PNRC interacts with the C-terminus, amino acids 212–316, of the human Pol III subunit RPC39. The yeast expression plasmids, pACT2-RPC39 and pACT2-RPC39_1–212_, for the expression of AD_Gal4_-RPC39 wild type (WT) or AD_Gal4_-RPC_1–212 _(1–212) fusion proteins were constructed as described in 'Methods'. The expression plasmids for AD_Gal4_-RPC39_212–316 _(212–316) and AD_Gal4_-RPC39_241–316_(241–316) were isolated from library screening through their interaction with PNRC_270–327_. Y187 cells were cotransformed with the expression plasmids coding for the fusion proteins as indicated. The selection of Y187 transformants and the β-Gal assays on transformants were performed as described in ***A***.

To identify additional proteins in breast tissue that interact with PNRC, we have screened mammary gland cDNA expression library twice with PNRC_270–327 _as bait using a Gal4-based yeast two-hybrid approach. The human PNRC_270–327 _yeast expression plasmid, pGBT9-PNRC_270–327_, was used to transform yeast strain CG1945. The CG1945 transformants bearing the pGBT9-PNRC_270–327 _plasmid were transformed again with a human mammary gland cDNA library (Clontech) DNA. Approximately 3.0 ~ 4.0 × 10^6 ^yeast transformants were screened in the absence of nuclear receptor ligands to identify additional PNRC-interacting proteins other than the nuclear receptors, known to interact with PNRC. A total of 22 His^+^/β-Gal^+ ^clones were isolated from these two screening. To our surprise, most of clones turned out to be PNRC itself, indicating that PNRC can form a dimer. Among the other PNRC-interacting clones, an RNA polymerase III subunit, RPC39, was found. RPC39_212–316 _and RPC39_241–316 _were isolated separately as PNRC_270–327_-interaction proteins in each screen. Human RNA polymerase III transcribes genes that produce small untranslated RNAs, including 5S RNAs, tRNAs, U6 RNA, 7SK RNAs, and viral associated RNAs such as adenovirus virus-associated (VA) RNAs. Human RNA pol III is composed of at least 16 subunits and three of these, hRPC32, hRPC39, and hRPC62, are specific for RNA pol III and can form a subcomplex that directs RNA Pol III binding to the TFIIIB-DNA complex via the interaction between TFIIIB and hRPC39 [5]. To test whether RPC39 truly interacts with PNRC and whether this interaction is specific for PNRC, independent yeast two-hybrid assays were performed to examine the interaction between RPC39_212–316 _and PNRC_270–327 _or other nuclear receptors and coactivators including PNRC2, SF1, ER/HBD, mERR3/HBD, and an irrelevant protein such as human lamin C protein (hLC). The expression of interacting hybrid proteins in yeast transformants were analyzed as *LacZ *expression. As shown in Fig. 1A, RPC39_212–316 _specifically interacts with PNRC_270–327_, but not with PNRC2, SF1, ER/HBD, mERR3/HBD nor an irrelevant protein, hLC, demonstrating that the interaction between RPC39_212–316 _and PNRC_270–327 _is specific.

### PNRC interacts with the C-terminus, amino acids 212–316, of the human RNA pol III subunit RPC39

The two RPC39 clones originally isolated from the yeast two-hybrid screening encoded two C-terminal peptides, aa 212–316 and aa 241–316, respectively. These two clones displayed similar abilities to interact with PNRC_270–327 _in yeast two-hybrid assays as shown in Fig. 1B, suggesting that RPC39 interacts with PNRC through its C-terminus. To test this hypothesis and to delineate more precisely the protein domains involved in the interaction between RPC39 and PNRC, the yeast expression plasmids for AD_Gal4_-RPC39 wild type or AD_Gal4_-RPC_1–212 _were constructed and tested in yeast two-hybrid assays for their interaction with DBD-PNRC_270–327_. As shown in Fig. 1B, the C-terminal of RPC39, aa 212–316 region, was found to interact as efficiently with PNRC_270–327 _as the wild type protein. In contrast, the N-terminal region of RPC39 (residues 1–212) did not detectably interact with PNRC. Therefore, RPC39 interacts with PNRC through its C-terminus.

### PNRC associates with RPC39 in mammalian cells

**Figure 2:**
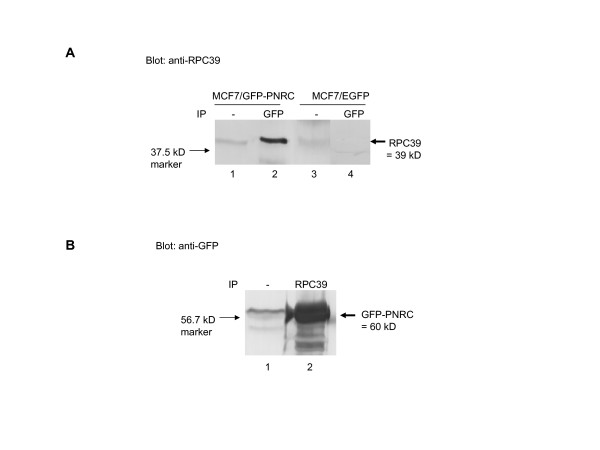
**PNRC associates with RPC39 in mammalian cells**. MCF-7/EGFP or MCF7/EGFP-PNRC stable expression cells were transiently transfected with the RPC39 expression plasmid, pSG5-RPC39, 24 h post-transfection, cells were harvasted and lysed, and 15 mg of total proteins were immunoprecipitated with antibodies against GFP (Clontech) or RPC39 (Santa Cruz Biotechnology). Specifically bound proteins to Protein A agarose beads were separated on a 10% SDS-PAGE and analysed by immunoblotting with either anti-RPC39 (***A***, blot: anti-RPC39) or anti-GFP (***B***, blot: anti-GFP) antibody, as previously described [2]. An aliquot of cell lysate equal to 100 μg of protein was included in each SDS-PAGE gel for Western blot to examine whether the crude protein extracts used for co-immunoprecipitation contains EGFP-PNRC and RPC39 proteins. The protein bands with molecular weights corresponding to those of RPC39 or EGFP-PNRC were indicated by arrows.

To confirm by an independent method that PNRC interacts with RPC39, we used coimmunoprecipitation assays to determine whether PNRC associates with RPC39 in mammalian cells. Due to a lack of commercial availability of PNRC antibody and the high background of PNRC antiserum generated in our laboratory, the MCF-7/EGFP-PNRC cells that stably express EGFP-PNRC fusion protein were used for this experiment. MCF-7/EGFP-PNRC cells or MCF-7/EGFP cells (as a control) were transiently transfected with the RPC39 expression plasmid, pSG5-RPC39. To determine whether PNRC associates with RPC39 in transfected MCF-7 cells, the EGFP-PNRC fusion protein was immunoprecipitated from MCF-7/EGFP-PNRC cell lysates with anti-GFP antibody and then the immunoprecipitates were analyzed with RPC39 antibody by Western blot. As shown in Fig. 2A, a protein with a molecular weight of 39 kDa was detected with the RPC39 antibody in the anti-GFP immunoprecipitates (Fig. 2A, lane 2) as well as in the crude protein extracts (lane 1) from MCF-7/EGFP-PNRC cell lysates by Western blotting. These results indicate that RPC39 protein coimmunoprecipitates with EGFP-PNRC fusion protein. There was no RPC39 band detected in the anti-GFP immunoprecipitates from MCF-7/EGFP control cell lysate (Fig. 2A, lane 4) although RPC39 was also expressed in the pSG5-RPC39 transfected MCF-7/EGFP cells as shown in Fig. 2A, lane 3 (Fig. 2A, lane 3), further demonstrating that RPC39 was associated with PNRC portion of EGFP-PNRC fusion protein, not EGFP portion of this fusion protein. To confirm the association of RPC39 and PNRC *in vivo*, we also immunoprecipitated RPC39 from the pSG5-RPC39 transfected MCF-7/EGFP-PNRC cell lysate with anti-RPC39 antibody and examined the presence of EGFP-PNRC protein in the RPC39-immunoprecipitates. Results in Fig. 2B illustrated that a protein with an apparent molecular weight of 60 kDa, which is close to the expected size of the EGFP-PNRC fusion protein (28+35 kDa), was detected in the anti-RPC39 immunoprecipitates (Fig. 2B, lane 2) as well as in the crude protein extract derived from the transfected MCF-7/EGFP-PNRC cells (Fig. 2B, lane 1), indicating that PNRC was coimmunoprecipitated with RPC39 by anti-RPC39 antibody. Therefore, the results from these experiments demonstrate that PNRC associates with RPC39 *in vivo*.

### PNRC stimulates RNA pol III-dependent transcription

**Figure 3:**
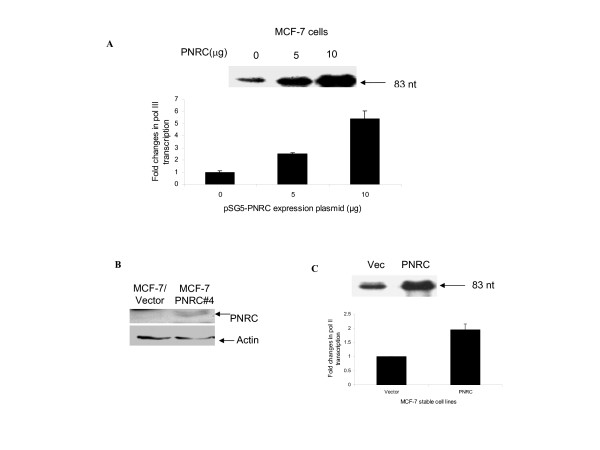
**PNRC stimulates the transcription of tRNA^arg ^gene by Pol III**. MCF7 breast cancer cells (***A***) were transiently transfected with 10 μg of reporter plasmid pArg-maxi which contains a Drosophila tRNA^arg ^gene and increasing amounts (0, 5, or 10 μg) of PNRC expression plasmid, pSG5-PNRC. Cells were cultured for 24 hours and the tRNA^arg ^transcripts in 1 μg of total RNA isolated from the transfected cells was determined by RNase protection assay using a ^32^P-labeled antisense riboprobe. An example of an autoradiogram of tRNA^arg ^transcript (indicated by an arrow) and quantification of the levels of tRNA^arg ^mRNA from three independent experiments are shown. ***B***, Western blot analysis of PNRC protein in MCF7/vector and MCF7/PNRC stably transfected cells. The procedures for transfection, G418 selection, and individual clone screening were described in Methods. An aliquot of cell lysate, from MCF7/vector or MCF7/PNRC cells, equal to 100 μg of protein was analysed by Western blot using PNRC antiserum (1:500 dilution) or anti-actin antibody (1:1000 dilution) as previously described [2]. The protein bands with molecular weights corresponding to those of PNRC or actin were indicated by arrows. ***C***, MCF-7/vector or MCF7/PNRC stable expression cells were transiently transfected with 10 μg of reporter plasmid pArg-maxi. Twenty-four hours after transfection, the tRNA^arg ^mRNA levels in the transfected cells were analysed by RNase protection assay as described for *A*. An example of an autoradiogram of tRNA^arg ^transcript (indicated by an arrow) and quantification of the levels of tRNA^arg ^mRNA from three independent experiments are shown.

Since hRPC39 is a specific and important subunit of human RNA pol III, it is tempting to speculate that the interaction of PNRC and RPC39 may play a role in the transcriptional regulation of genes transcribed by RNA pol III/RPC39. To test this hypothesis, we sought to determine the influence of PNRC overexpression on the transcription of RNA pol III transcribed target genes. We first transiently transfected MCF-7 cells with a reporter tRNA gene plasmid to measure RNA pol III transcription in the presence of increasing amounts of PNRC expression plasmid, pSG5-PNRC. tRNA^arg ^transcripts were detected from the transfected cells by RNase protection assay using a ^32^P-labeled antisense riboprobe. As shown in Fig. 3, a dose-dependent increase in tRNA gene transcription was observed upon the introduction of increasing amounts of PNRC expression plasmid (Fig. 3A). To confirm this result, we then constructed stable cell lines expressing increased PNRC protein, named MCF-7/PNRC cells. As shown in Fig. 3B, the level of PNRC protein in MCF-7/PNRC cells was much higher than that in the MCF-7 cells transfected with vector alone (MCF-7/Vector). These stable cell lines were transiently transfected with the tRNA gene reporter. Results in Fig. 3C illustrated that tRNA gene transcription in the PNRC stable expression MCF-7 cells was much higher than that in the control vector transfected stable MCF-7 cells, supporting that overexpression of PNRC, either by transient transfection or stable transfection, stimulates the transcription directed by RNA pol III.

### Down regulation of RNA pol III-dependent transcription by specific depletion of endogenous PNRC using RNA interference

**Figure 4:**
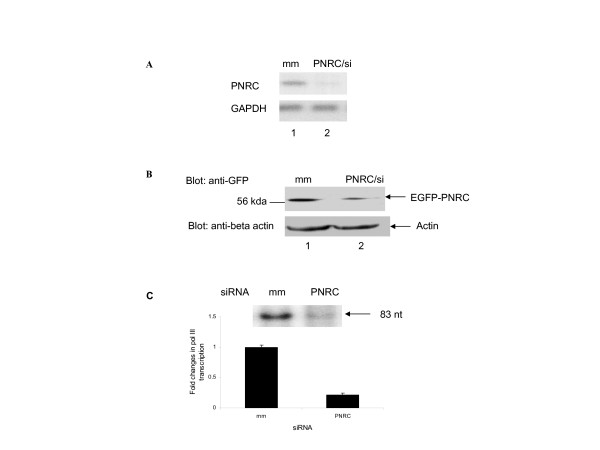
**Down regulation of RNA Pol III gene transcription by specific depletion of endogenous PNRC using RNA interference**. ***A***, Northern blot analysis of PNRC and GAPDH mRNAs. MCF7 cells were transiently transfected with Pol III reporter plasmid, pArg maxi, along with 20 nM of either siRNA specific for PNRC (PNRC/si) or nonspecific mismatch RNA (mm). Twenty-four hours after transfection, 20 μg of total RNA, isolated from the transfected cells, was subjected to Northern analysis with PNRC and GAPDH cDNA probes, separately, as described in Methods section. ***B***, Western blot of PNRC and actin protein. An aliquot of cell lysate equal to 100 μg of protein prepared from PNRC/siRNA (20 nM) transfected (PNRC/si) or nonspecific mismatch RNA (mm) transfected MCF7/EGFP-PNRC cells was separated on a 10% SDS-PAGE and subjected to Western analysis with 1:1000 diluted anti-GFP mouse monoclonal antibody (Cloetech) and anti-actin antibody (Santa Cruz Biotechnology) separately. The protein bands with molecular weights corresponding to those of EGFP-PNRC fusion protein or actin were indicated by arrows. ***C***, Down regulation of tRNAarg transcription by PNRC/siRNA. MCF-7 cells were transiently transfected with reporter plasmid, pArg maxi (10 μg) along with 20 nM of either PNRC/siRNA (PNRC) or mismatch control RNA (mm). Twenty-four hours after transfection, the levels of tRNA transcripts in the transfected cells were determined by RNase protection assay as described in figure 3.

To further determine whether PNRC is a true activator of RNA pol III, the influence of down regulation of PNRC on transcription directed by RNA pol III in MCF-7 cells was examined. MCF-7 cells were transfected with either siRNA specific for PNRC (PNRC/siRNA) or nonspecific mismatch RNA (mm). As shown in Fig. 4A, PNRC mRNA was decreased dramatically by PNRC siRNA (lane 2) but not by the introduction of nonspecific mismatched RNA (lane 1). As shown in Fig. 4B, EGFP-PNRC fusion protein was decreased significantly upon the treatment of PNRC siRNA (Fig. 4B, lane 2). The down regulation of PNRC by PNRC/siRNA is specific as the level of GAPDH mRNA (Fig. 4A, lower panel) and the level of actin protein (Fig. 4B, lower panel) in the cells did not change upon the treatment of PNRC/siRNA. By knocking down the expression of PNRC in MCF-7 cells, PNRC/siRNA treatment also remarkably reduced RNA pol III-transcribed tRNA gene transcription by 5 fold (Fig. 4C). Together, these results support that PNRC stimulates RNA polymerase III-dependent transcription.

### PNRC and RPC39 are co-recruited onto RNA polymerase III-dependent genes

**Figure 5:**
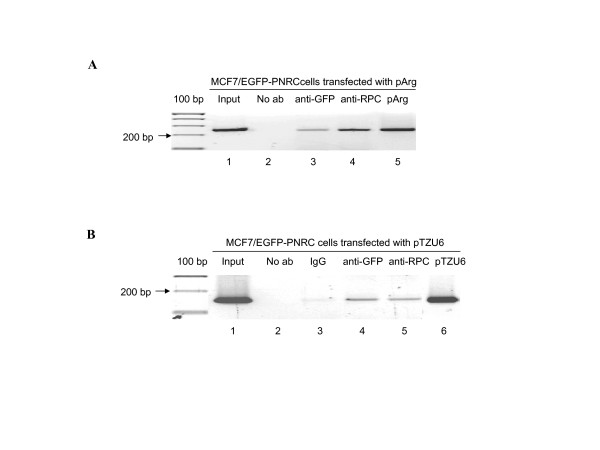
**Co-recruitment of PNRC and RPC39 onto RNA polymerase III-dependent genes**. MCF-7/EGFP-PNRC cells were transfected either with pArg maxi reporter plasmid, which contains tRNA^arg ^gene promoter and coding sequence, or with pTZU6 plasmid that carries the U6 RNA gene promoter. Twenty-four hours after transfection, cells were treated with formaldehyde to crosslink endogenous proteins and DNA. Samples of sonicated and purified chromatin were immunoprecipitated with no antibody (No ab), preimmuno IgG (IgG), GFP antibody (anti-GFP), or RPC39 antibody (anti-RPC) as indicated. DNA isolated from immunoprecipitated material was amplified by PCR with primers to amplify a 220 bp fragment of the Drosophila tRNA^arg ^gene (***A***) or a 160 bp fragment of the U6 RNA gene (***B***). The amplified PCR products were analyzed on 1.8% agarose gel. The PCR products from the reactions using input DNA, pArg or pTZU6 plasmids as template were included on the gels as positive and size controls.

The above experiments have demonstrated an interaction between PNRC and RPC39 and the biological relevance of this interaction in regulating transcription of genes transcribed by RNA pol III. These results suggest that PNRC might be bound to these genes. To test this hypothesis, we employed chromatin immunoprecipitation (ChIP) analysis to examine the promoter occupancy of PNRC and RPC39 on tRNA^arg ^gene and U6 RNA gene promoters. MCF-7/EGFP-PNRC cells were co-transfected with either the tRNA^arg ^gene or the U6 RNA gene, and the expression plasmid for RPC39, pSG5-RPC39. MCF7/EGFP-PNRC cells were chosen in this experiment because GFP antibody could be used to precipitate EGFP-PNRC bound promoter regions of the RNA pol III transcribed genes. Results in Fig. 5A showed that the tRNA^arg ^gene was only amplified from the precipitates by anti-GFP (Fig. 5A, lane3) or anti-RPC39 (Fig. 5A, lane 4) antibody. Only a very weak band was amplified in the PCR reaction with the precipitated DNA from the no antibody control reaction (Fig. 5A, lane 2). To validate that the DNA fragment amplified was the expected 220-bp region of the tRNA^arg ^gene, this fragment was subcloned into TA cloning vector and then sequenced. Sequence data confirmed that the PCR product has the sequence of tRNA^arg ^gene (data not shown). Similar results were obtained from the PCR analysis of chromatin immunoprecipitates using the primer set for the amplification of the U6 RNA gene (Fig. 5B). Together, these results demonstrate recruitment of PNRC and RPC39 to the genes transcribed by RNA pol III.

## Discussion

In order to further characterize the role of PNRC in gene activation, we sought to clone cellular proteins other than the nuclear receptors that specially interact with the C-terminus of PNRC harboring an SH3-binding motif. The yeast two-hybrid interaction screen identified RPC39, an RNA pol III-specific subunit, as one potential partner of PNRC. Several independent methods confirmed the interaction and association of PNRC and RPC39 *in vitro *(Fig. 1) and *in vivo *(Fig. 2). Chromatin immunoprecipitation assays revealed the presence of PNRC at tRNA and U6 RNA gene promoters in MCF-7 cells (Fig. 5). Accordingly, tRNA gene transcription was induced when exogenous PNRC was introduced into MCF-7 cells by either transient transfection (Fig. 3A) or stable transfection (Fig. 3C). This effect is not just a feature of PNRC overexpression. Specific depletion from MCF-7 cells of endogenous PNRC using RNA interference caused a five-fold decrease in tRNA gene transcription (Fig. 4C). These results suggest that PNRC can function as a modulator of RNA pol III transcription by directly interacting with RPC39 subunit.

There are three RNA polymerases in eukaryotic cells, with distinct functions. Each is responsible for synthesizing a different set of products. RNA pol I synthesizes the large rRNAs, RNA pol II synthesizes mRNAs and some untranslated RNAs, whereas RNA pol III transcribes many untranslated RNAs including tRNA, 5S rRNA, and U6 RNA. Human RNA pol III is the largest RNA polymerase with the largest number of subunits [6]. It is composed of at least 16 subunits and three of these, hRPC32, hRPC39, and hRPC62, are specific for RNA pol III and play important roles in accurate transcription initiation [5,7]. RNA pol III plays a key role in cell growth control by catalyzing the production of tRNA and 5S rRNA. Although this is often regarded as a housekeeping function, it is in fact highly regulated [8]. RNA pol III transcription increases markedly when mammalian cell pass through the G1/S transition [9,10]. Many types of transformed cell lines have been shown to overexpress RNA pol III products. Growth factors trigger a rapid increase in RNA pol III activity and this is essential for cell proliferation [11]. The tumor suppressors, p53 and RB, have been demonstrated to repress RNA pol III transcription through their interaction with TFIIIB [8,12-15], while oncoproteins, including Ras [16], c-Myc [17], E7 [18,19], and E1A [20,21], strongly stimulate RNA pol III transcription by activating TFIIIB directly or indirectly.

After cloning of the first authentic steroid receptor coactivator 1 (SRC-1) in 1995 [22], more than 50 nuclear receptor cocativators have been cloned [23]. In general, coactivator proteins do not bind to DNA, but interact indirectly through association with other DNA-binding proteins such as nuclear receptors. Hormone binding triggers nuclear receptor translocation and the release of the corepressor complex and subsequent recruitment of nuclear receptor coactivator to the target gene promoters. Once recruited to the promoter, coactivators enhance RNA pol II transcription activity through a combination of mechanisms, including efficient recruitment of basal transcriptional factors. In addition, nuclear receptor coactivators possess themselves, or recruit other nuclear proteins that possess enzymatic activities crucial for efficient gene expression including the acetyl-transferase (CBP/p300 [24], pCAF [25], and p160s [26,27]), methyltransferases (CARM1 [28], PRMT-1 [29], and PRMT2 [30]) and ubiquitin ligases (E6-AP [231 and Rsp5 [32]]). It has been reported that p160 nuclear receptor coactivator such as GRIP1 only selectively stimulates the transcription of RNA pol II but not promoters transcribed by RNA pol III [33]. Previously, we demonstrated that PNRC interacts with nuclear receptors and functions as a coactivator to enhance transcription by RNA pol II, mediated by multiple nuclear receptors [2]. Here, we demonstrate that PNRC also stimulates transcription by RNA pol III through its interaction with the RNA pol III-specific subunit, RPC39. This finding makes PNRC a unique coactivator.

## Materials and methods

### Cell lines and reagents

MCF-7 cells, human breast adenocarcinoma cells, were purchased from ATCC and were grown in Eargle's MEM supplemented with 10% fetal bovine serum, 100 IU of penicillin per ml, 100 μg of streptomycin per ml and nonessential amino acid and sodium pyruvate. For the co-immunoprecipitation experiment and down-regulation of tRNA^arg ^transcription by PNRC/siRNA experiment, MCF-7/EGFP-PNRC cells, which stably express the EGFP-PNRC fusion protein, were used. The culture condition was the same as that for MCF-7 cells except that 1 mg/ml of G418 was included in the culture medium.

The MATCHMAKER Two-Hybrid System kit including a human mammary gland MATCHMAKER cDNA library was purchased from Clontech (Palo Alto, CA). Yeast transformation kit was purchased from Bio 101 (La Jolla, CA), and the yeast culture media were purchased from Clontech. Various restriction endonuclease were purchased from New England Biolabs (Beverly, MA). Hot start Taq polymerase was purchased from Qiagen (Valencia, CA). siRNA for PNRC was purchased from Ambion (Austin, Texas). Anti-RPC39 mouse monoclonal antibody and anti-actin were obtained from Santa Cruz Biotechnology, Inc. (Santa Cruz, CA). Anti-GFP antibody was purchased from Clontech. Anti-PNRC antiserum was generated as previously described [4]. Lipofectamine 2000 was purchased from Invitrogen Corporation (Carlsbad, CA). ExpressHyb solution was purchased from Clontech. [α-^32^P dCTP] was purchased from ICN Biomedicals, Inc. (Irvine, CA) and the Megaprime DNA labeling kit was obtained from Amersham Biosciences (Piscataway, NJ). Oligonucleotide primers were synthesized in the DNA/RNA chemistry laboratory at the City of Hope. DNA sequencing was done in City of Hope Sequencing Core facility.

### Construction of plasmids

All recombinant DNA and plasmid constructions were prepared according to standard procedures, and the sequences and orientations of inserted DNA fragments in plasmid constructs were verified by standard DNA sequencing. Yeast expression plasmids for DBD_Gal4_-PNRC_270–327 _or DBD_Gal4_-PNRC fusion proteins, named pGBKT7-PNRC_270–327 _or pGBKT7-PNRC, were prepared by inserting PCR-amplified coding regions for PNRC_270–327 _or for the full length PNRC into pGBKT7 vector through *Nde*1 and *Sal*1 sites, respectively. The plasmid for the expression of DBD_Gal4_-PNRC2 was prepared by inserting the PCR-amplified PNRC2-coding region into pGBKT7 vector through the *Nde*1 and *Sal*1 sites. The construction of the plasmids for the expression of DBDGal4-SF1, DBDGal4-hER/HBD, and DBDGal4-mERR3/HBD were previously described [2,3].

The expression plasmids for the expression of ADGal4-RPC39 and ADGal4-RPC39_1–211 _were generated as follows. The coding regions of RPC39 and RPC39_1–211 _were amplified by PCR using pOTB7/RPC39 plasmid (Invitrogen) as template. The primers for PCR amplification of RPC39 coding sequence are 5'GCCGAATTCCCATGGCGGAGGTGAAGGTG-3' and 5'-CGGCTCGAGCTAAAATTCGAGCCACTCTGTCAT-3' and the primers for PCR amplification of RPC391-211 coding region are 5'-GCCGAATTCCCATGGCGGAGGTGAAGGTG-3' and 5'CGGCTCGAGCTAGGCA-AATGAACTATTTCT-3'. The *EcoR*1 and *Xho*1 digested PCR products were then inserted into pACT2 vector at *EcoR*1 and *Xho*1 sites.

The GFP-PNRC expression plasmid (i.e. pEGFP-PNRC) was constructed by inserting the human PNRC coding region into the *Bgl *II/*Sal *I sites at the 3' end of the coding region of the GFPmut1 variant in the pEGFP-C1 vector (Clontech Laboratories, Inc.). The expression plasmid for PNRC-V56His was generated using Echo Cloning System (Invitrogen) that quickly and easily recombines a gene of interest into a series of recipient (acceptor) vector. Briefly, the coding region for wild type PNRC was amplified by PCR using pUC13-B4-2 [34] as template and forward primer, 5'-GCCCTCGAGATGACTGTCGTCTCCGTCCCG-3', and reverse primer, 5'-AATGAGCTCAGTTTGAACTTTGAGGAGGGT-3'. The PCR product was purified by agarose gel electorphoresis, digested with *Xho*1 and *Sac*1, and subcloned into a donor vector, pUni/V5-HisA (Invitrogen) through *Xho*1/*Sac*1 sites so that V5-6His is placed at the C-terminus of PNRC. The recombinant donor plasmid, pUni-PNRC-V56His, was then mixed with the adaptor vector, pcDNA3.1E (Invitrogen), in the presence of Cre recombinase. The recombination reaction mixture was then transformed into TOP10 *E. coli *and the recombinants were selected by plating the transformation reaction onto plates containing kanamycin. The transformants were analyzed for recombinant plasmid, pcDNA3.1E/pUni-PNRC-V56His, by restriction digestion and sequencing. The RPC39 expression plasmid in mammalian cells, pSG5-RPC39, was generated by inserting the PCR-amplified RPC39 coding region into pSG5 vector at *EcoR*1/*Bgl *II sites.

### Yeast two-hybrid screening

The yeast MATCHMAKER two-hybrid system (Clontech) was used to screen a MATCHMAKER mammary gland cDNA expression library according to the supplier's protocol (Clontech protocol PT1030-1) using pGBKT7-PNRC_270–327 _as the bait. Briefly, the bait plasmid, pGBKT7-PNRC_270–327_, was transformed into yeast reporter stain CG1945 along with a human mammary gland MATCHMAKER cDNA expression library (Clontech) in the Gal4 activation domain vector (pACT2, Clontech). Transformants (3.42 × 10^6^) were screened first for HIS3 reporter gene expression by plating them onto plates lacking histidine, leucine, and tryptophan, and His^+^/Leu^+^/Trp^+ ^transformants were recovered and further screened for β-galactosidase activity using the colony-lift filter assay according to the supplier's protocol. Plasmids of both His^+ ^and β-galactosidase positive transformants were isolated from yeast. The nucleotide sequences of inserts in true positive hybrid plasmids were generated, and the identity of the cDNAs was determined through a homology search against known sequences in GenBank.

### Yeast two-hybrid assay to detect the interaction between two known proteins

Yeast two-hybrid assay was used to examine protein-protein interaction *in vivo*. Briefly, the yeast strain Y187 was co-transformed with the AD vector alone or AD-RPC39 wild type or its fragments along with pGBT9 (DBD_Gal4 _vector only), pGBT9-/target plasmids (for PNRC 270–327, SF1, ER/HBD, and mERR3/HBD) or DBD-hLC negative control plasmid. The transformants that carry both plasmids were selected by plating them onto SD/-Leu/-Trp plates and were further analyzed for β-galactosidase activity by liquid β-galactosidase activity measurement as essentially described in the protocol. Briefly, the transformants that grew on SD/-Leu/-Trp agar plates were cultured overnight in liquid SD/-Leu/-Trp medium at 30°C. This overnight culture was diluted in liquid YPD and then continued to incubate for four to six hours. At the end of this incubation, a liquid β-Galactosidase activity assay, using ONPG as a substrate, was performed according to the manufacturer's protocol (Clontech).

### Western blot and coimmunoprecipitation

Western analyses were done as previously described [2]. The immunoprecipitation experiments with antibodies against GFP (for the detection of EGFP-PNRC fusion protein) or RPC39 antibody were performed using an immunoprecipitation kit (protein A) (Roche Molecular Biochemicals, Mannheim, Germany) according to the manufacturer's instructions. Briefly, the lysis buffer (ice cold, 50 mM Tris-HCl, pH 7.5, 150 mM NaCl, 1% Nonidet P40, 0.5% sodium deoxycholate, and the complete set of protease inhibitor cocktail) was added to the PBS-washed cells. After 20 minutes of incubation on ice, the cells were then scraped and transferred into Eppendorf tubes, sonicated, and centrifuged at 12,000 × g at 4°C for 10 minutes. The amount of cell lysate used for immunoprecipitation was 15 mg. To reduce background, aliquots of cell lysats were mixed with 50 μl of the homogeneous protein A agarose suspension and incubated at 2~8°C overnight on a rocking platform. The agarose beads were removed by centrifugation at 12,000 × g at 4°C for 20 seconds. The supernatant was then incubated with the specific antibodies against GFP (1:1000) or RPC39 (1: 500 dilution) and gently rocked for 1 hour at 4°C. A 50 μl of the homogeneous protein A agarose suspension was added and incubated further at 2~8°C overnight on a rocking platform. The complexes were collected by centrifugation at 12,000 × g at 4°C for 20 seconds. The precipitates bound beads were washed each time using washing buffer 1 (same as lysis buffer), washing buffer 2 (ice cold, 50 mM Tris-HCl, pH 7.5, 500 mM NaCl, 0.1% Nonidet P40, 0.05% sodium deoxycholate) and washing buffer 3 (ice cold, 10 mM Tris-HCl, pH 7.5, 0.1% Nonidet P40, 0.05% sodium deoxycholate). The bead bound precipitates were eluted with gel loading buffer, separated on a 12% SDS-PAGE, and detected by Western blotting, using either the GFP antibody or RPC39 antibody. To check whether EGFP-PNRC and RPC39 expressed in the cells, 100 μg of lysate was also included in each corresponding gel for Western analysis. The immuno-protein complex was detected by a 1:1000 diluted goat anti-rabbit horseradish peroxidase conjugate (Pierce Chemical Co.) followed by the DAB (diaminobenzidine tetrahydrochloride, Sigma) substrate visualization.

### Establishment of PNRC stable expression MCF-7 cell lines

MCF-7 cells in 6-well plate were transfected with 5 μg of either expression vector pEGFP-C1 (Clontech) or pcDNA3.1E (Invitrogen) or expression plasmids for either EGFP-PNRC fusion protein (pEGFP-PNRC) or PNRC-VA6His (pcDNA3.1E-PNRCVA6His) using Lipofectamine 2000. At 48 h after transfection, the transfected cells were split in fresh medium containing G418 (1 mg/ml) at 25% confluent and were cultured in this selection medium continuously until G418-resistant foci can be identified (for about three weeks). The G418 resistant, pEGFP or pEGFP-PNRC transfected MCF-7 cells were pooled and further selected by flow cytometry cell sorting on the base of their GFP fluorescence. For pcDNA3.1E or pcDNA3.1-PNRC-V56His transfected MCF-7 cells, the individual G418-resistant foci was picked and expanded in 24-well plates. Individual G418 resistant clones were screened for the highest level of PNRC expression by Northern blot, Western blot, and PNRC coactivation function analysis.

### RNase protection assay

RNase protection assays were carried out basically as previously described [35]. Briefly, MCF-7 cells were cotransfected with the expression plasmid for PNRC, pSG5-PNRC, and pArg-maxi gene for 24 h using Lipofectamine 2000. In some cases, the MCF7/PNRC cells that stably express PNRC were transfected with only the pArg-maxi gene for 24 h. For examining the effect of PNRC siRNA on Pol III transcription, MCF-7 cells were co-transfected with pArg-maxi gene along with either mismatch RNA or siRNA specific for human PNRC gene. Total RNA was isolated from the transfected cells by Trizol reagent (Invitrogen). RNase protection assays were carried out as described in the protocol of Ambion, Inc. The ^32^P-labeled riboprobe for tRNA^arg ^gene was prepared as follows. The pArg-maxi gene plasmid was digested with *Xba*1, and labeled RNA probe was synthesized using the MAXIscript T7 kit (Ambion) with [^32^P]CTP. Total RNA (1 μg) isolated from the transfected cells was hybridized with the labeled tRNA^arg ^RNA probe and incubated at 40°C overnight. The samples were digested with RNase A/T1 at 37°C for 30 min and precipitated. The samples were incubated on dry ice for 15 min and centrifugated at 14, 000 rpm for another 15 min. The pellets were resuspended in gel loading buffer (Ambion) and separated on 8% acrylaminde-8 M urea denaturing gels. The bands were visualized by autoradiography, and the RNA products were quantified by Phosphorimager.

### PNRC/siRNA transfection and cell proliferation analysis using RT-CES

The purified and annealed PNRC siRNA with the target sequences of 5'-GGAAAGAGUUUUAAAAUCtt (sense) and 5'-GAUUUUAAAACCUCUUUCCtg (antisense) was purchased from Ambion. MCF-7 or MCF-7/EGFP-PNRC cells in 6 well plates were transfected with 2 μl of siPORT Lipid (Ambion) alone (mock transfection) or together with 20 nM of PNRC siRNA or control siRNA, GAPDH negative control siRNA, according to the manufacturer's instruction. At 8 h, 24 h or 48 h after transfection, cell lysate (100 μg) or total RNA (20 μg), prepared from the transfection cells, was subjected to Western blot as previously described [2] or Northern analysis as described below.

### Northern hybridization

Total RNA was isolated from MCF7 cells using TRIzol reagent (Invitrogen). Twenty μg total RNA was analyzed by Northern hybridization. After being fractionated on a 1.0% agarose/formaldehyde gel by electrophoresis, RNA was transferred overnight in 10× SSC onto a Zeta Probe GT membrane (Bio-Rad) and the membrane was baked at 80°C for 1 hour. A 740-bp human PNRC cDNA was generated by *Eco*R1 restriction digestion of pSG5-PNRC plasmid and gel purification. 100 ng of this DNA fragment was radioactively labeled with [α-^32^P] dCTP (ICN) using the Megaprime DNA labeling kit (Amersham Biosciences) and used as probe in hybridization at the concentration of 2 × 10^6 ^cpm/ml. The membrane was wet in 5 × SSC before prehybridized in ExpressHyb solution (Clontech) at 68°C for 30 min and hybridized at 68°C for 1 hour. The membrane was then rinsed twice with 2× SSC/0.05% SDS and washed twice, each 15 min, with same buffer at room temperature. If necessary, the membrane was further washed with 0.1 × SSC/0.1% SDS at 50°C for 5–15 min and exposed to X-ray film (Kodak) overnight at -70°C. After stripping off the PNRC2 probe, by washing in the boiling 0.5% SDS for 10 min at room temperature, the membrane was hybridized with a ^32^P-labeled 840-bp human GAPDH cDNA probe, generated by RT-PCR, using the following forward and reverse primers: 5'-ACA GCC GCA TCT TCT TGT GCA GTG-3' and 5'-TCA GAT GCC TGC TTC ACC ACC TTC-3'.

### Chromatin immunoprecipitation assay (ChIP)

ChIP assay was performed using ChIP Assay kit (Upstate) according to the manufacturer's instructions. Briefly, approximately 10^6 ^of MCF-7/RGFP-PNRC cells cultured in 10 cm dish were transiently transfected with pArg maxi (contains tRNA^arg ^gene) or pTZU6 (contains U6 RNA gene). Twenty-four hours after transfected, cells were treated with 1% formaldehyde (final concentration, v/v) in cell culture medium for 10 min at 37°C to cross-link proteins to DNA. The cells were washed twice with phosphate-buffered saline, pH 7.4 containing protease inhibitor cocktail (Roche Molecular Biochemicals), harvested by centrifugation and resuspended in 200 μl SDS lysis buffer (1% SDS, 10 mM EDTA, 50 mM Tris-HCl (pH 8.1), and protease inhibitor cocktail) and incubated on ice for 10 min. Samples were sonicated on ice with a Digital Sonifier (Dranson Ultrasonics Corporation, CT) at 30% of maximum power 10 times for 15 s each, with 2 min of cooling on ice in between sonications.

The sonicated DNA was diluted 10-fold in ChIP dilution buffer (0.01% SDS, 1.1% Triton X-100, 1.2 mM EDTA, 16.7 mM Tris-HCl (pH8.1), 167 mM NaCl, and protease inhibitor cocktail). A fraction of the diluted supernatant, 1%, or ~20 μl, was transferred to a new tube and kept as input for PCR. The rest of the supernatant was pre-cleared by incubation with protein A Sepharose beads for 2 hours at 4°C. The antibodies against GFP (for EGFP-PNRC fusion protein) or RPC39 (2 μg each) were then added and the samples were incubated overnight at 4°C. For a negative control, a no antibody immunoprecipitation or normal rabbit IgG immunoprecipitation was also performed. Immunocomplexes were precipitated for 2 hours with protein A Sepharose beads and the precipitates were washed once with 1 ml of low salt wash buffer (0.1% SDS, 1% Triton X-100, 2 mM EDTA, 20 mM Tris-HCl (pH 8.1), and 150 mM NaCl), once with 1 ml of high salt wash buffer (0.1% SDS, 1% Triton X-100, 2 mM EDTA, 20 mM Tris-HCl (pH 8.1), and 500 mM NaCl), once with LiCl wash buffer (0.25 M LiCl, 1% IGEPAL-CA630, 1% deoxycholic acid (sodium salt), 1 mM EDTA, 10 mM Tris, pH 8.1), and twice with TE buffer. All washes were for 5 min, rotating at 4°C. The protein/DNA complexes were eluted from beads with 250 μl of elution buffer (1% SDS, 0.1 M NaHCO_3_). Formaldehyde cross-links were reversed by overnight incubation at 65°C and the proteins subsequently degraded by proteinase K. DNA was recovered by phenol-chloroform extraction and ethanol precipitation in the presence of 0.3 M sodium acetate and 20 μg glycogen. PCR amplification of a 220 bp of the Drosophila tRNA^arg ^gene was performed using a sense primer 5'-ATG TAC ACT AGA GTA GTA TCC ACA TAT-3' and an anti-sense primer 5'-CTA GCT AGT TGA TGC ACT GCT TGG AGG-3' [36]. PCR amplification for a 160 bp fragment of U6 RNA gene was also performed using a forward primer 5'-GTACAAAATACGTGACGTAGAAAG-3' and a reverse primer 5'-GGTGTTTCGTCCTTTCCAC-3' [37]. HotStarTaq DNA polymerase (Qiagen) was used to perform PCR utilizing the following conditions: Initial denaturation for 15 min at 95°C followed by 40 cycles of denaturation at 95°C for 1 min, annealing at 55°C for 1 min, extension at 72°C for 1 min ending with a final 7 min extension at 72°C. The PCR products were resolved electrophoretically on a 1.6% agarose gel and visualized with EtBr staining. The PCR products were further subcloned into TA cloning vector (Invitrogen) and sequenced.
